# Soil salinity and aridity specify plague foci in the United States of America

**DOI:** 10.1038/s41598-020-63211-4

**Published:** 2020-04-10

**Authors:** Rémi Barbieri, Gaëtan Texier, Catherine Keller, Michel Drancourt

**Affiliations:** 1 Aix-Marseille Univ., IRD, MEPHI, IHU Méditerranée Infection, Marseille, France; 2 Aix Marseille Univ., CNRS, EFS, ADES, Marseille, France; 3 Aix Marseille Univ., IRD, AP-HM, SSA, VITROME, dIHU-Méditerranée Infection, Marseille, France; 4 Centre d’épidémiologie et de santé publique des armées [CESPA], Marseille, France; 5 Aix Marseille Univ., CNRS, IRD, INRAE, Coll. France, CEREGE, Aix-en-Provence, France

**Keywords:** Ecological epidemiology, Soil microbiology

## Abstract

Plague is a deadly zoonosis that periodically reemerges as small outbreaks in geographically limited foci where the causative agent *Yersinia pestis* may reside in soil. We analyzed a dataset of 1.005 carefully documented plague cases that were georeferenced over 113 years in peer-reviewed literature in the contiguous United States. Plotting outbreaks by counties defined as plague foci on geographical maps, we observed a significant co-localization of plague outbreaks with high soil salinity measured by an electric conductivity of >4 dS/ m^−1^ and aridity measured by an aridity index <0.5. Thus, we identified aridity and soil salinity as significantly associated with ecological risk factors for relapsing plague in the contiguous United States. These results reveal two evolutive parameters that are partially associated with anthropic activities, complicating the epidemiology of plague in the contiguous United States. Exploiting aridity and soil salinity data may help in the surveillance of evolving plague foci in the contiguous United States.

## Introduction

Plague is a deadly zoonosis caused by the bacterium *Yersinia pestis*. This bacterium evolved among ancient human populations, as revealed by the genome sequencing of causative *Y. pestis* strains from Neolithic farmers^1^ and Bronze Age individuals in Eastern and Central Europe^2–4^. Two notable historical plague pandemics swept over Europe, disseminating primarily along coastal, maritime trade routes and terrestrial pilgrimage routes. The Justinian plague spanned from 541 to 750/767, while the Black Death occurred from 1347 until the mid-18th century^5–9^. The current third pandemics began with the microbiological era after Alexandre Yersin obtained the first ever *Y. pestis* isolate in 1894^10^. Plague is currently a deadly World Health Organization notifiable disease^11^ (only for pneumonic forms and/or non-endemic cases) that has caused 2.886 new cases, including 504 deaths, between 2013 and 2018^12^.

During inter-zoonotic periods, plague dissemination is restricted to so-called plague foci, i.e., restricted to geographic areas where plague sporadically reemerges, increasing its dissemination among susceptible animals and further promoting its dissemination among human populations^13,14^. Plague foci have been reported in specific areas in Eurasia, Asia, Africa and Americas^15–41^, including Madagascar and the Democratic Republic of Congo, two countries with the largest numbers of plague cases over the past ten years, as illustrated by the 2017 epidemic that caused 171 deaths in Madagascar^42,43^. In these foci, plague is thought to be maintained by a subtle interplay between plague-susceptible and plague-resistant small mammals and their ectoparasites^13,39^. Furthermore, our recent study confirmed that *Y. pestis* resides in soil in plague foci in Algeria, suggesting that interactions between mammals and contaminated soil may play a role in maintaining plague foci^44^. These observations were reinforced by experimental evidence of the one-year survival of virulent *Y. pestis* in soil^45^ and field observations, culminating with the isolation of the fourth telluric *Y. pestis* strain that we named Algeria3^44^. This first ever soil isolate from Africa followed three isolates being obtained from Honk-Kong^10^, Iran^46^ and Grand Canyon Park, Arizona^47^. Comparing the *Y. pestis* Algeria3 genome with 133 other *Y. pestis* genomes resulted in this strain being biotyped as a biovar *Y. pestis* Orientalis. In the Algerian plague foci, we demonstrated that a 0.5–70 g/L NaCl in soil, was one factor contributing to the persistence of *Y. pestis* in soil^44^.

We questioned whether these observations were restricted to one plague focus in Algeria or could occur in other parts of the world. We chose to investigate this possibility in the contiguous United States (USA) because it is the only country where plague has been carefully and continuously investigated since its introduction in 1900, resulting in the production of high quality, exhaustive data for analyses. Furthermore, most of the historical data having been anonymized by aggregation at the county level, fulfilling rules of confidentiality. The purpose of this study was to investigate potential relationships between population-level exposure to risk factors such as soil salinity and aridity and plague cases.

## Results

**Figure 1:**
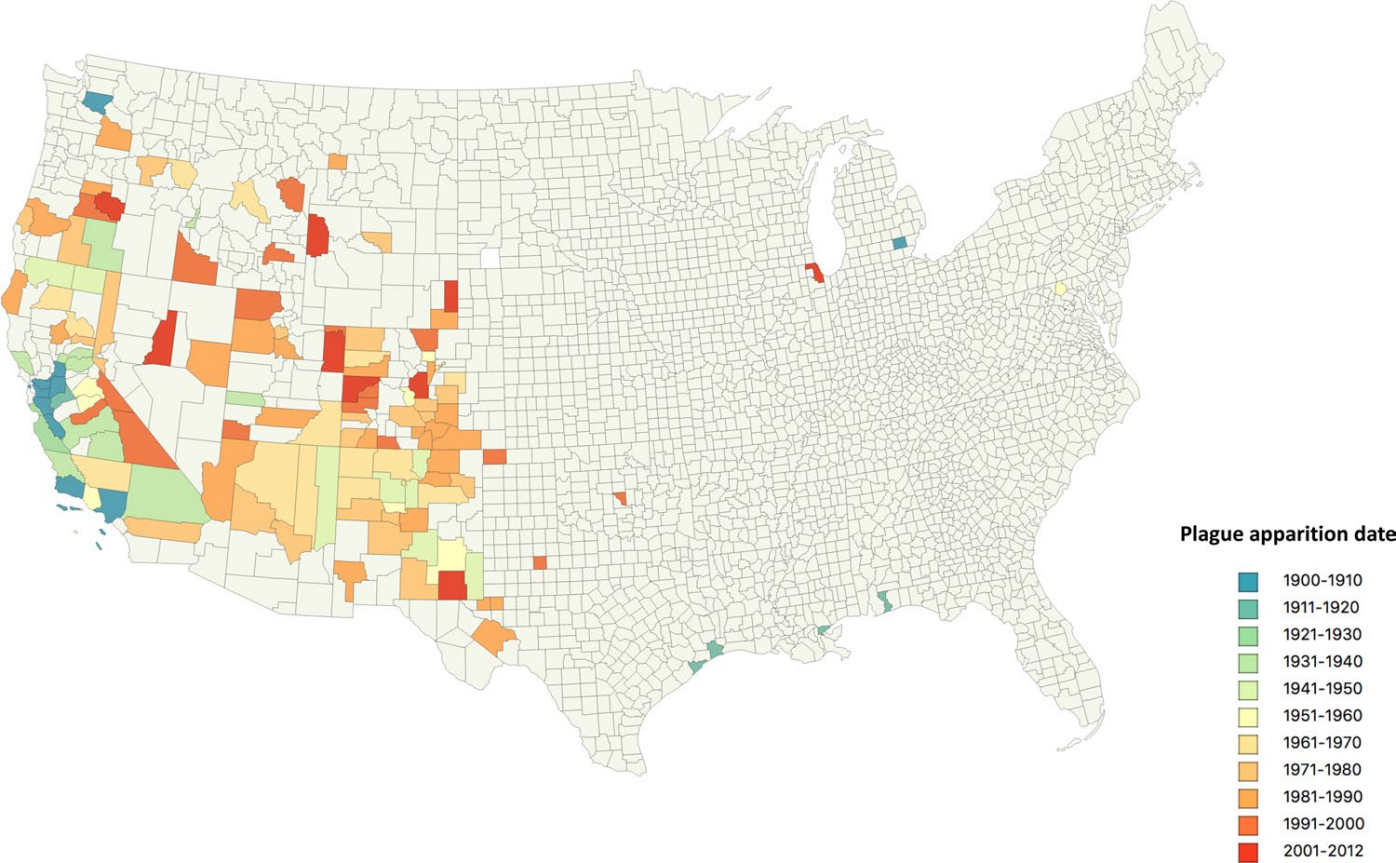
Dynamic of plague in the USA, 1900 to 2012: The map features the appearance of the very first case per county, corresponding to 136 counties. Color code corresponds to the chronology.

In this study, 1.005 plague cases reported in the USA from 1900 to 2012 and further compiled by Kugeler *et al*.^48^ (see the “plague cases and plague foci” section below) were localized to 136 counties in the USA. To understand the dynamics of the appearance of plague in counties, we plotted the first case registered in all the 136 plague-affected counties on a map where the color scale features the time scale of first registration (from blue to red using a 10-year time scale except for the twelve years 2001 to 2012 (time scale, 12 years) (Fig. 1). The very first case of plague recorded in the USA was reported in March 1900 in San Francisco, California, by the bacteriologist W.H. Kellog, who performed the autopsy of a Chinese man. The plague rapidly spread, claiming 21 deaths the same year. We observed that from 1900 to 1910, all cases were located in the West Coast, primarily in ports, including San Francisco, Santa Barbara, Los Angeles and King (Washington) counties. An exception to this pattern was the occurrence of one case due to the accidental contamination of a scientist handling *Y. pestis* in a laboratory in Washtenaw county, Michigan^49^. From 1911 to 1920, plague spread in several California counties and reached ports of the Mexican Gulf, including Galveston and Jefferson (Texas), New-Orleans Parish (Louisiana) and Escambia (Florida) counties. From 1921 to 1930, plague was endemic in California and spread to the south and east of the state. In the 1930s, plague northeast to Lake county (Oregon) and Gem county (Idaho) and east to Beaver county (Utah). From 1941 to 1950, the plague migrated further east, reaching the arid Great Plains of Arizona and New Mexico, and in the 50s, new cases were documented in Northern New Mexico in Colorado (Boulder and Chaffee counties). From 1960 to 2000, the number of plagues affecting counties increased by a factor of 2.5 compared to 1900–1960. Cases were then concentrated in southwest in Utah, Colorado, Arizona and New Mexico. During the last decade, plague spread north to Wyoming, northwest to Oregon and west to Nevada. Two cases documented in Illinois and Michigan were traced to manipulations of *Y. pestis* by laboratory workers^49,50^, while a third case was documented in Frederick county, Maryland, at the Army’s Biological Warfare Headquarters^51^.

**Figure 2:**
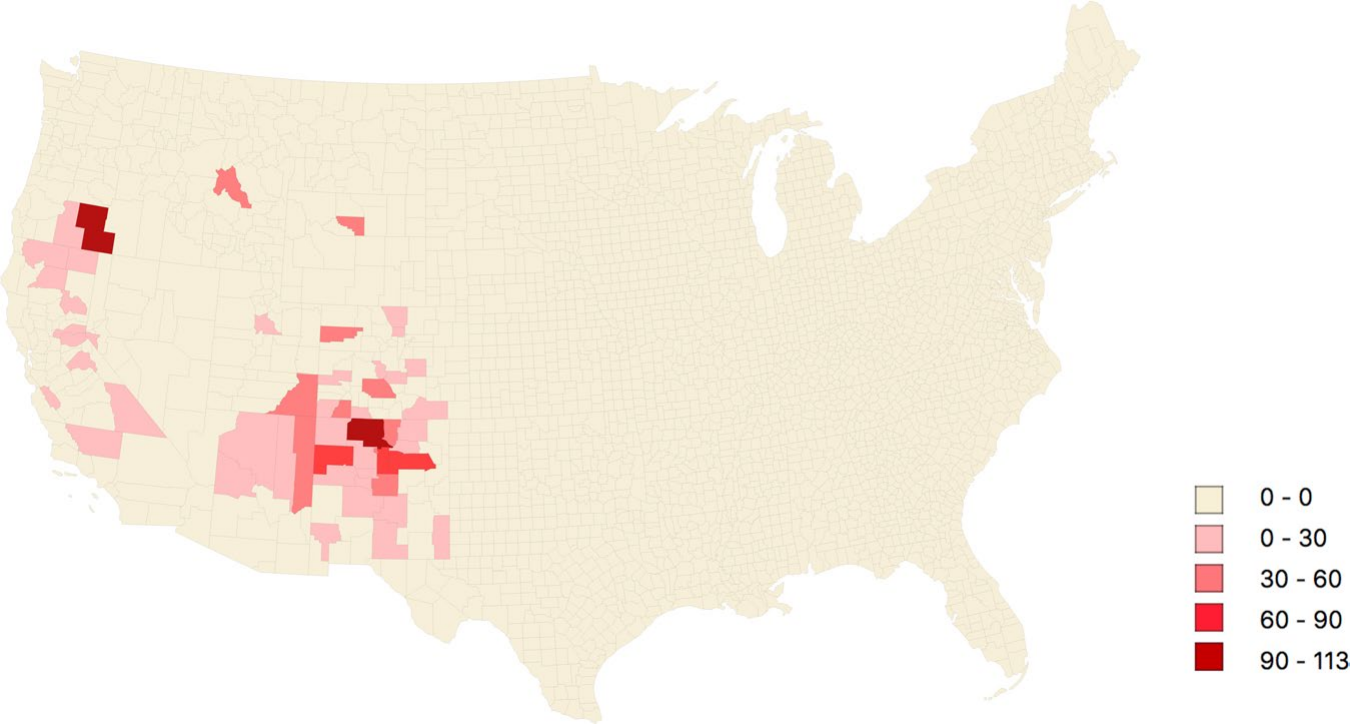
Choropleth map of the 57 plague foci featuring cumulative incidence per 100,000 inhabitants per county in the USA, 1910–2012.

**Figure 3:**
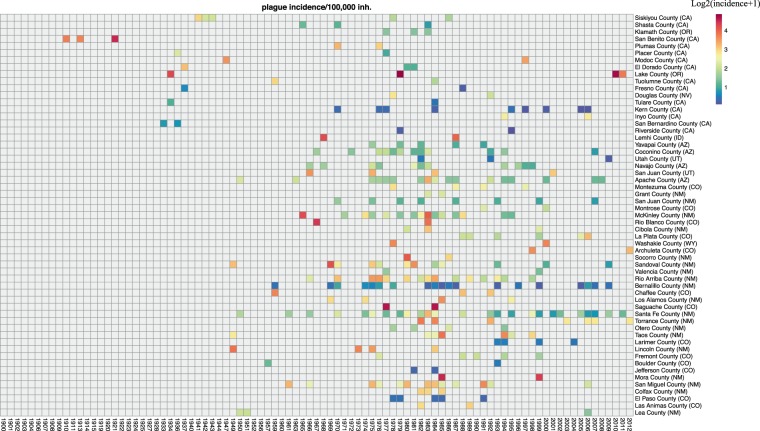
Heatmap of yearly plague incidence in 57 plague foci (x axis) classified from western counties to eastern counties (y axis) in the USA, 1910–2012.

We then analyzed all 1.005 plague cases registered over 113 years in the USA, plotting them on a series of 11 choropleth maps featuring the yearly plague incidence per county (Supplementary Fig. 1). Each map represents a 10-year time period except for the twelve years 2001 to 2012 (time scale, 12 years). We observed that California was the only state to continuously experience cases from 1900 to 2012, with a total of 34 outbreaks and 459 cases, making California the state with the highest number of cases. Oregon reported plague for the first time in 1934, and after being absent for three decades, plague reemerged in 1970 and was continuously reported until 2012. Since the 1940s, plague was also reported in Utah, Colorado, Arizona and New Mexico, with 64 plague outbreaks reported for a total of 401 plague cases. After defining a plague focus as a county where at least two cases were reported at least one year apart (see the “plague cases and plague foci” section below), we identified 433 plague cases distributed into 297 outbreaks and 57 foci (counties) representing approximately 7% of the total surface of the USA. Plotting these 433 cases onto a choropleth map yielded the cumulative plague incidence per 100,000 inhabitants per focus (Fig. 2). To visualize the temporal distribution of plague foci over 103 years (from 1910 to 2012), we distributed these 433 cases on a heatmap featuring the annual incidence in the 57 plague foci (Fig. 3). These 57 plague foci were located within nine western states, including New Mexico (19 counties), California (14 counties), Colorado (13 counties), Arizona (four counties), Oregon (two counties), Utah (two counties), Nevada (one county), Idaho (one county) and Wyoming (one county). Two counties (Lake county, Oregon, and Rio Arriba county, New Mexico) presented a high incidence of greater than 90 cases per 100.000 inhabitants (Supplementary data 3). We noted that 88% of plague foci were located in California, Arizona, New Mexico and Colorado. In New Mexico, Arizona, California and Colorado, 57, 26, 24, and 20% of counties were plague foci, respectively.

Subsequently, we plotted aridity data on a USA county map and observed that longitude 98°W separated all of the 661 (100%) arid counties located in the western USA from the 2995/3.106 (96%) non-arid counties located in the eastern USA (*p* < 10^−5^) (Supplementary Fig. 2). Indeed, 111 non-arid counties are located in the western USA, primarily in the Northwestern USA, and 25 non-arid counties are located along the Pacific Ocean coast from the state of Washington to Southern California. Three states are completely arid, including Nevada, Arizona and New Mexico. In total, arid counties represent 20.5% of all the USA counties. We also plotted salinity data on the USA county map and observed that saline counties are concentrated in Midwestern and Southwestern USA (Supplementary fig. 3). The entire surface of Arizona and Nevada contain saline soil, except for the Storey county in Nevada. We counted 410 saline counties (12.8% of counties) in the USA, including 200 counties located west of longitude 98°W and 210 counties located east.

**Figure 4:**
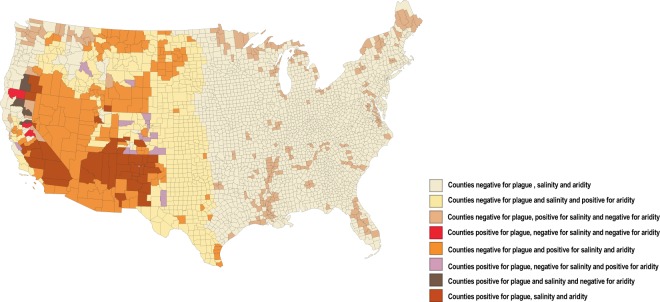
Cumulative map featuring aridity, salinity and the 57 plague foci per county in the USA.

**Figure 5:**
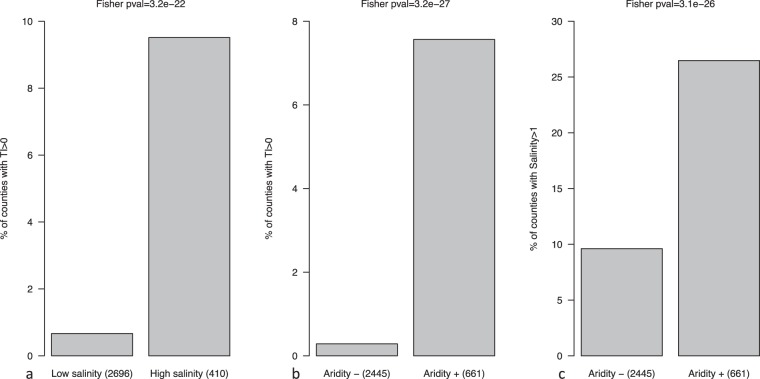
Fisher’s exact testing the association of plague in the 57 plague foci versus salinity (**a**), aridity (**b**) and salinity versus aridity (**c**) in the USA.

Finally, we plotted aridity and salinity data and plague foci together on a USA map of counties classified into eight categories (Fig. 4). One geographical area located in Northern New Mexico, representing 26% of all plague foci, combines aridity and plague foci, while an area in Northern California and Southern Oregon, representing 7% of all plague foci, combines saline soil and plague foci. Finally, one area located in Southern California, North Arizona, New Mexico, Oregon, Utah, Colorado, Wyoming and Nevada, representing 67% of all plague foci, combines plague, aridity and salinity, and in these counties, we observed a significant statistical association between plague, aridity and salinity (*p* < 10^−5^). These observations suggest a link between soil salinity and aridity in one particular county and the presence of plague foci in the same county. Accordingly, we noticed a statistically significant association between plague foci and saline counties (*p* < 10^−5^) odd ratio (OR) = 15.6 [8.9; 27.6]; (Fig. 5a) and between plague foci and arid counties (*p* < 10^−5^) OR = 28.5 [12.9–63.2]; (Fig. 5b). Moreover, we identified a significant link between aridity and saline counties (*p* < 10^−5^) OR = 3.4 [2.7; 4.2] (Fig. 5c).

**Figure 6:**
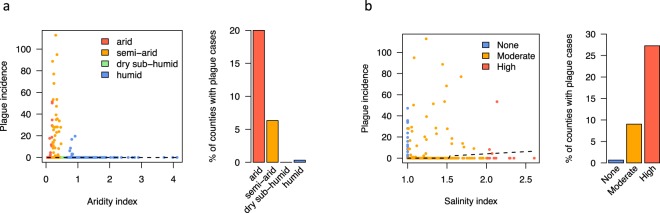
Ecological studies of cumulative plague incidence per 100,000 inhabitants in the 57 plague foci versus aridity (**a**) (expressed as mean Aridity Index by county (see material and methods)), and salinity (**b**) (expressed as mean Salinity index by county (see material and methods)) per county in the USA. The dotted black lines represent fitted Loess regressions. On the right of each scatterplots, the percentage of counties in which plague cases were documented is reported using barplots.

Subsequently, we used an ecological study to analyze a putative quantitative link between aridity, salinity and plague. We plotted aridity versus plague incidence, then soil salinity versus plague incidence and also, we fitted a Loess regression to identify a possible trend between plague incidence and soil status (Fig. 6). Aridity is probably a preliminary condition for plague (426/433–98% plague cases occurred in counties with an aridity rate <0.5; only seven cases occurred in counties presenting an aridity rate between 0.5 and 1). Although there was no linear relationship between the aridity index and the plague incidence rate, plague incidence rates are significantly associated with arid and semi-arid counties (Fisher’s exact test pval = 3.2e-27, Figs. 5b and 6a). Similarly, high salinity levels were associated with increased plague incidence rates (Figs. 5a and 6b). A negative binomial analysis was performed with times of exposition 1 (TE1) spanning between the first outbreak until the last one; and time of exposition 2 (TE2) spanning between the first outbreak until 2012. TE1 and TE2 indicated a significant link between plague reemergence and aridity: TE1, OR = 1.94 [1.14–3.62] TE2, OR = 2.58 [1.13–5.94]; and a significant link between plague reemergence and salinity: TE1, OR = 1.73 [1.24–2.50]; TE2, OR = 1.87 [1.10–3.25].

These calculations confirmed that salinity and aridity are two significant factors associated with the reemergence of plague in affected counties in the USA.

## Discussion

In this study, we observed that plague foci documented in the USA for 103 years significantly associated with aridity and soil salinity. Specifically, we demonstrated that high level of aridity and soil salinity were significantly associated factors for the local resurgence of human plague. Interestingly, a previous study established that plague outbreaks in the pre-industrial Europe were often preceded by arid episodes, which is consistent with our results^52^. These observations add to the understanding of telluric plague in plague epidemiology, after several strains of *Y. pestis* have been recovered from soil samples collected on three continents^10,44,46^. In the USA, one *Y. pestis* strain was from the soil collected beneath an approximately 3-week old carcass of a plague-infected mountain lion in Grand National Canyon Park, Mohave county, Arizona^47^. The Mohave county is one of 38 counties in the USA exhibiting a high rate of aridity and soil salinity (salinity = 1.3; PET = 0.14) (Supplementary figs. 2 and 3), where two human plague cases were registered in 1988 and 2007^47^. In some cases, the edaphic persistence of *Y. pestis* at a shallow depth of 4–15 centimeters^10,47^ may be facilitated by the occurrence of a “leaching” zone generated periodically by rain (or irrigation) and allowing *Y. pestis* to move downwards with water and salt and then move upwards during the dry periods^53^. The interpretation of the data reported here should be forecasted by the complex, multifactorial ecology of plague. One possibility is that soil aridity /salinity governs the repertoire of animal species such as rodents typically found in arid and semi-arid areas, acting as plague sources^54^. In this study, 75% of plague foci collocated with the habitats of rodent species known to be sources of plague, i.e., the black tailed prairie dog, Gunnison’s prairie dog and the black footed ferret in New-Mexico and Arizona and the Gunnison’s and black and white-tailed prairie dog and black footed ferret in Colorado, Kansas and Nebraska^14^. These rodents generally live in wide open xeric grassland characterized by a shallow soil^55^. In the USA, the seasonality of epizooties is a matter of debate. Some epizooties occur during the rainfall season, when humidity favors the reproduction and development of fleas and soil moisture favors the development of numerous healthy rodents through better feeding conditions^14^, as illustrated by epizootic plague among black-tailed prairie dog populations correlating with soil exhibiting a high moisture-holding capacity^56^. Alternatively, some epizooties occur during the dry season with scarce precipitation or just after rainfall when the temperatures become warmer, with all of these conditions favoring flea hatching and abundance^57,58^. Altogether, our results might reconcile these two observations whereby arid, high salinity soils - which are known to favor water retention during rainfall season or irrigation^59^ - might acquire high moisture-holding capacities following rare rainfall episodes and further support plague spreading via subsequent seasonal epizooties. These aridity and soil salinity conditions probably determine changes in ecosystems influenced by global warming and anthropic activities, including deforestation, soil over-exploitation^60^, soil irrigation with saline water or used waters and/or crops poor drainage^59^. Although salinity was estimated at the county level, the data may lack accuracy, as regional scale soil salinity evaluation remains a challenge^61^. In addition, salinity mostly studied because of its negative impacts on agriculture^62^, has progressively spread as a consequence of improper management of irrigation in arid areas or crop-fallow systems encouraging soil moisture storage (as in Montana^63^). Irrigated areas tend also to increase to meet the increasing food and fiber demand^64^, as in the USA^65^, together with the associated secondary salinization. Evolving aridity and soil salinity could contribute to spread plague foci, as observed in the USA over the past century.

In conclusion, our study identified aridity and soil salinity as key ecological determinants of the natural history of plague in the USA; pointing to foci worth of microbiological investigations to balance the relative role of soil and animals in the preservation of plague; guiding active surveillance of sentinel mammals.

## Materials and Methods

### Plague cases and plague foci

We collected plague data used in the study of Kugeler *et al*.^48^ (courtesy of the Centers for Disease Control, Atlanta, Georgia; raw data may be available from CDC’s Division of Vector-Borne Diseases upon request) recorded in the USA over 113 years, including the very first case of plague reported in San Francisco in 1900 up to 2012. We collected data on 1.005 plague cases that fulfilled at least one of the three diagnostic criteria previously defined by Kugeler *et al*.^48^. These 1.005 cases were georeferenced over 136 different counties exclusively located in the contiguous U.S. (Supplementary Fig. 1). We then defined counties in which two plague cases have been reported spanning at least a one-year period of time as being *bona fide* plague foci. Putative imported cases related to the transit of travelers and/or infected merchandise were not considered to limit bias due to non-native cases. Accordingly, we excluded plague cases diagnosed in counties bordering the ocean and/or including port cities. Finally, we used 433 plague cases distributed in 57 plague foci spread over 57 counties from 1910 to 2012 in the USA (Fig. 2).

### Aridity data

Global aridity datasets were based on a study by Antonio Trabucco^66^. Aridity was expressed using aridity index (AI) which was calculated as the reported mean annual precipitation divided by the mean annual potential evapotranspiration (PET). Mean annual precipitation values were obtained for the years 1950–2000 from the WorldClim Global Climate Data^67^. Layers estimated on a monthly average basis by the Global-PET (i.e., modeled using the Hargreaves method) were aggregated to generate mean annual values. Global aridity was visualized as a grid layer world map in 30 arc second resolution, where each pixel represents the annual AI average over the 1950–2000 period. AI was classified into five different classes according to the United Nations Environment Programme:^68^ 1) <0.03, hyper arid 2) 0.03–0.2, arid; 3) 0.2–0.5, semi-arid; 4) 0.5–0.65, dry sub-humid and 5) <0.65, humid. In this study, the mean aridity was calculated considering all the values represented by each pixel within a county and considered counties to be arid (englobing semi-arid area because evaporation is bigger than precipitation) where the mean AI was less than or equal to 0.5. Using these criteria, we identified 661 arid counties that were evaluated in this study (Supplementary fig. 2).

### Salinity and sodicity data

We used a World map Database of excess of salt and sodium in soil from Food and Agriculture Organization^69^ where soil salinity is defined by electric conductivity values higher than 4 dS m^−1^ in some horizons within 100 cm of the soil surface; or defined by sodicity, which is defined by more than 6 percent saturation of exchangeable sodium in the cation exchange capacity in some horizons within the first 100 cm (also referred to as exchangeable sodium percentage, ESP). After the extraction of soil salinity/sodicity data for each county, we input to each county the mean of salinity/sodicity calculated considering all the values represented by each pixel (on a 1–4 Salinity index^69^ in which; 1 = non-saline soil, 2 = moderate saline soil, 3 = high saline soil and 4 = very high saline soil) within a county and defined a saline county having a mean soil salinity greater than 1. Using this criterion, we identified 410 saline counties that were evaluated in this study.

### Plague incidence rate

The plague incidence was calculated for each county using a population estimation at the year of plague appearance per county. County population data were retrieved from the United State Census Bureau (USCB), with census data collected every ten years from 1900 to 2010. We estimated the population every ten years using the population growth rate between each census. The population estimation for the year 2011 was based on the population growth rate between 2000 and 2010. The 2012 population estimation was based on a projection by the USCB. In total, 403 population estimations were calculated for counties over 103 years and 297 annual incidence rates of plague (Supplementary data 1 and 2). Figure 2 was modelled from a cumulative rate of plague incidence per 100,000 inhabitants per county from 1910 to 2012. For this reason, we evaluated 57 cumulative incidence rates spread over 57 plague foci in the USA in this study (Supplementary data 3).

### Statistical and GIS analyzes

All statistical analyses were performed using R 3.5.0^70^. All remote sensing image manipulations, data and buffer extraction, geographical analysis and maps were performed using QGIS^71^.

We used Fisher’s exact test to compare qualitative variable and calculated Odd ratio (OR) with its confidence interval at 95% to quantify strength of association between soil characteristics, aridity and plague.

We removed from the regression analysis (which attempts to describe a possible link between exposition and disease) all of the counties that did not fulfill the following criteria: plague-free county where no previous case/foci of plague occurred (necessarily having a non-zero probability to observe the case/foci), and for salinity exposure analysis, we also removed all counties with an average soil saline mean per county equal to 1, which represents a non-saline soil (necessarily having a non-zero probability to observe the exposition). We then generated on each plot a linear regression and a non-linear and non-parametric local regression. To generate a Loess curve (robust locally weighted), at each point in the range of the data set, a low-degree polynomial was fitted to a subset of the data^72^. Because plague is rare (requiring aggregation of data for any analysis to be carried out), and because measurements at an individual level are not always available (most of historical data were anonymized by aggregation at a small area level (the county), cases of surveillance data requiring confidentiality), we decided to perform an ecological study^73^. The purpose of our study was to investigate the possible relationship between population-level exposure to risk factors (salinity, aridity) and disease (plague cases).

Primary effects of covariates (salinity, aridity) on the number of plague foci were assessed using a negative binomial regression. For this analysis, we assessed three variables for each of the 57 plague foci: (1) the number of outbreaks (one outbreak is defined by a higher number of cases than expected by year), (2) the mean of aridity and (3) the mean of salinity. We fitted two different models using 2 offsets variables (indicating the amount of time that each count is based on) Time of exposition 1 [TE1] = time spanned between the first outbreak until the last one; and Time of exposition 2 [TE2] = time spanned between the first outbreak until 2012.

## Supplementary information

Supplementary figures 1–3.

Supplementary datasets 1–3.
