# Supplementary material for: Soil salinity and aridity specify plague foci in the United States of America
Source: Sci Rep. 2020 Apr 10;10:6186. doi: 10.1038/s41598-020-63211-4 (PMC7148359; doi:10.1038/s41598-020-63211-4)
Supplement: Supplementary file 1 — Supplementary figures 1–3. [file 41598_2020_63211_MOESM1_ESM.pdf]

Soil salinity and aridity specify plague foci in the United States of America.

Rémi Barbieri[a,b], Gaëtan Texier[c,d], Catherine Keller[e], Michel Drancourt[a*]

a. Aix-Marseille Univ., IRD, MEPHI, IHU Méditerranée Infection, Marseille, France.

b. Aix Marseille Univ., CNRS, EFS, ADES, Marseille, France.

c. Aix Marseille Univ., IRD, AP-HM, SSA, VITROME, IHU-Méditerranée Infection, Marseille, France.

d. Centre d'épidémiologie et de santé publique des armées [CESPA], Marseille, France.

e. Aix Marseille Univ., CNRS, IRD, INRA, Coll. France, CEREGE, Aix-en-Provence, France.

* Corresponding author : Michel Drancourt

IHU Méditerranée Infection, 19-21 Bd Jean Moulin 13005 Marseille,

France. Tel: +33 (0)4 13 73 24 01 fax: + 33 (0) 13 73 24 02.

Email: Michel.Drancourt@univ-amu.fr

SUPPLEMENTARY FIGURES

Supplementary Figure 1

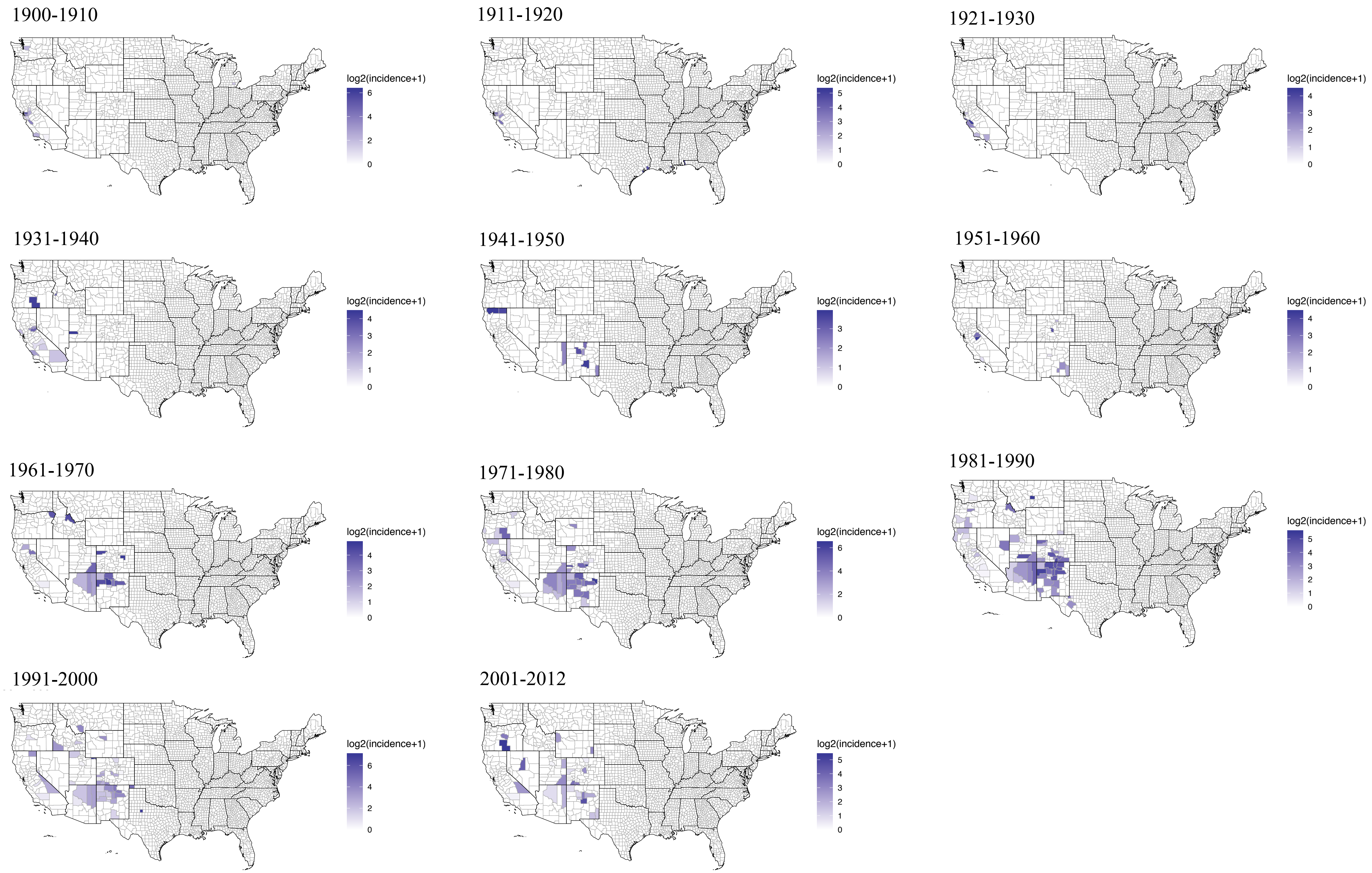

Supplementary figure 1. Choropleth maps of the all 1.005 plague cases documented from 1900 to 2012 per county in contiguous USA.

Supplementary figure 2

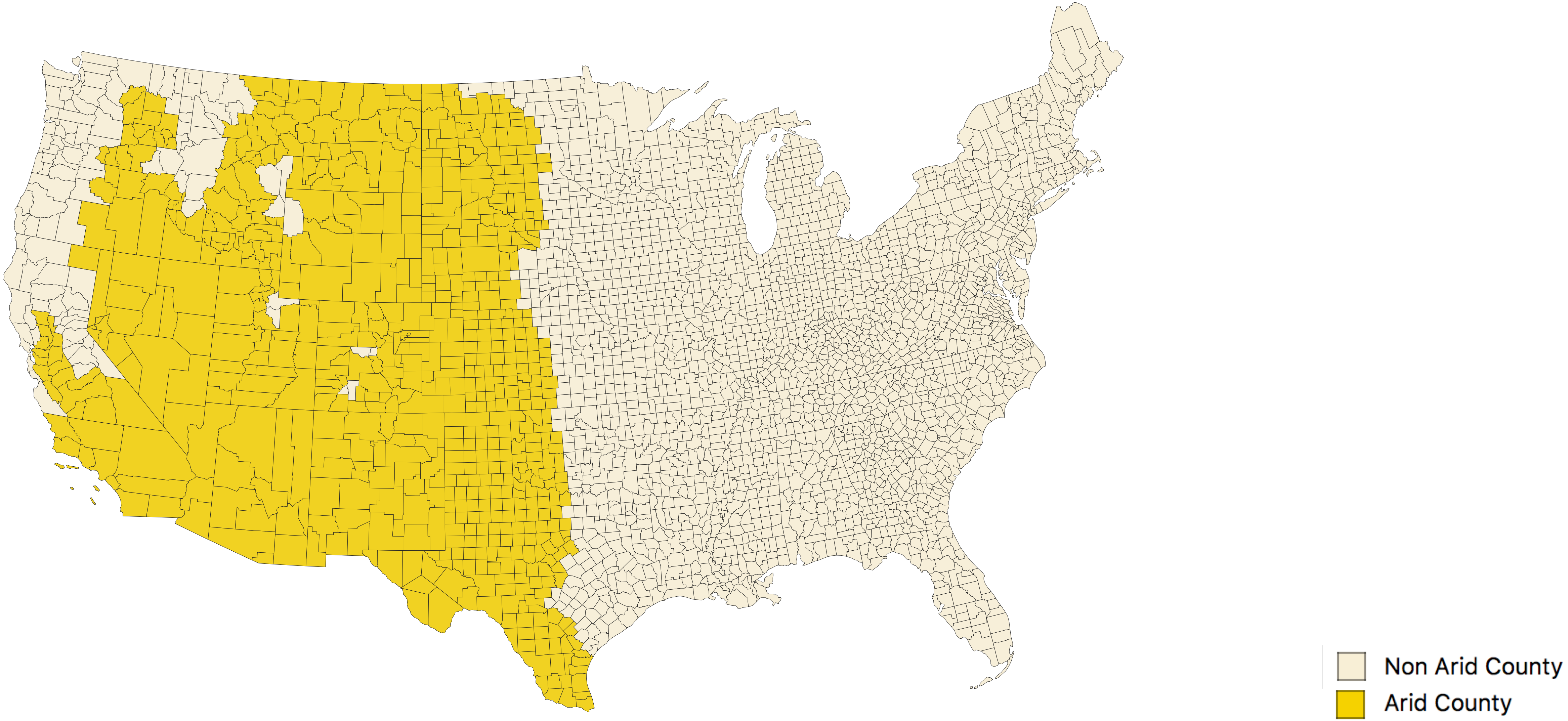

Supplementary figure 2. Map of aridity per county in contiguous USA.

Supplementary figure 3

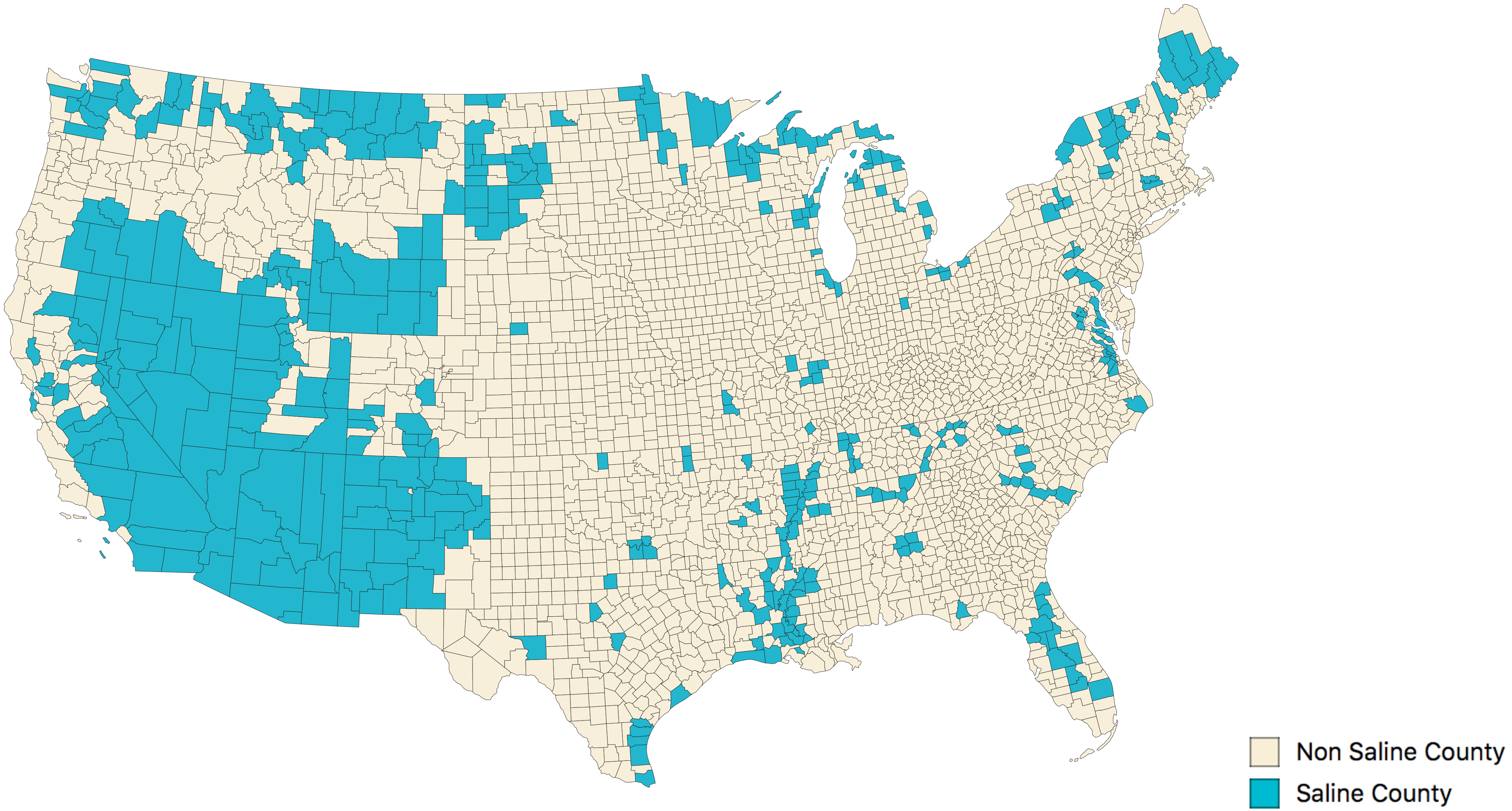

Supplementary figure 3. Map of salinity per county in contiguous USA.

Supplementary data legends

Supplementary data 1. Estimations of population in the 57 plague foci over 103 years. These estimates were performed only for plague years in contiguous USA.

Supplementary data 2. Plague's rate of incidence per year in the 57 plague foci in contiguous USA.

Supplementary data 3. Plague cumulative incidence in the 57 plague foci in contiguous USA.
